# Monoamine oxidase B levels are highly expressed in human gliomas and are correlated with the expression of HiF-1α and with transcription factors Sp1 and Sp3

**DOI:** 10.18632/oncotarget.6582

**Published:** 2015-12-12

**Authors:** Martyn A. Sharpe, David S. Baskin

**Affiliations:** ^1^ Department of Neurosurgery, Kenneth R. Peak Brain and Pituitary Tumor Center, Houston Methodist Hospital, Houston, TX 77030, USA

**Keywords:** MAOB, glioma, HiF-1α, Sp3, Sp1

## Abstract

Monoamine oxidases A and B (MAOA and MAOB) are highly expressed in many cancers. Here we investigated the level of MAOB in gliomas and confirmed its high expression. We found that MAOB levels correlated with tumor grade and hypoxia-inducible factor 1-alpha (HiF-1α) expression. HiF-1α was localized to the nuclei in high-grade gliomas, but it was primarily cytosolic in low-grade gliomas and normal human astrocytes. Expression of both glial fibrillary acidic protein (GFAP) and MAOB are correlated to HiF-1α expression levels. Levels of MAOB are correlated by the levels of transcription factor Sp3 in the majority of GBM examined, but this control of MAOB expression by Sp3 in low grade astrocytic gliomas is significantly different from control in the in the majority of glioblastomas. The current findings support previous suggestions that MAOB can be exploited for the killing of cancer cells. Selective cell toxicity can be achieved by designing non-toxic prodrugs that require MAOB for their catalytic conversion into mature cytotoxic chemotherapeutics.

## INTRODUCTION

The current treatment modalities for glioblastoma multiforme (GBM) are palliative rather than curative [1]. Following initial diagnosis, patients receiving maximal surgical resection, concomitant radiotherapy and chemotherapy with temozolomide, and additional adjuvant temozolomide have an average survival of 16.9 months [2, 3]. Temozolomide is an orally active alkylating agent and its cytotoxicity is a function of its ability to methylate DNA; however, this damage can be repaired by the enzyme *O*^6^-methylguanine DNA methyltransferase (MGMT). In patients treated with temozolomide, the median survival of those with background levels of MGMT expression is 21.7 months, but this falls to 12.7 months in patients where the MGMT promoter region is methylated [4].

Monoamine oxidase (MAO, EC 1.4.3.4) catalyzes the oxidative deamination of monoamine neurotransmitters such as serotonin, noradrenaline, dopamine and phenylethylamine. Two isoenzymes, MAOA and MAOB, have been identified in humans; both are present on the outer mitochondrial membrane, but each isoenzyme has different substrate and inhibitor sensitivities. There are considerable differences in distribution of MAOA and MAOB in tissues, in cell-types, in enzyme activity and in substrate kinetics in mammals [8]. The specific activity of MAOB is higher in the primate brain than in other organs, such as the kidneys, liver, heart, and lungs [9, 10]. In the normal human adult brain, MAOB is found in serotonergic neurons and in non-neuronal cells, such as astrocytes and radial glia [11–13].

It has been reported that compared to normal brain tissue, MAOB activity is significantly greater in tissue from GBM, low-grade astrocytomas, and anaplastic astrocytomas, but but meningioma tissue shows no increase in MAOB activity with respect to control brain tissue [14].

Hypoxia-inducible factor 1 (HIF-1α) initiates the transcription of a number of hypoxia-inducible genes, including those encoding vascular endothelial growth factor and its receptors. High levels of nuclear localized HIF-1α are observed in the majority of glioblastomas and anaplastic astrocytomas, particularly surrounding areas of necrosis in glioblastomas [15], and expression levels correlated with gliomal tumor grade [16, 17].

GFAP levels in astrocytic tumors correlate with tumor grade and lethality [18, 19], and serum levels of GFAP and GFAP-positive circulating tumors cells are diagnostic of tumor grade and lethality [20–22]. In mild ischemia of the brain there is an upregulation of astrocytic HIF-1α and GFAP and upregulation of GFAP is blocked by HIF-1α inhibitor YC-1 [23]. Mild hyperthermia has been demonstrated to upregulate GFAP expression in cultured astrocytes and it appears that increased GFAP expression results from increases in nuclear HIF-1α levels [24]. In astrocytic chemical models of hypoxia, exposure to CoCl_2_ increases HIF-1α, NF-κB and GFAP levels [25]. This CoCl_2_ driven GFAP expression is sensitive to the Rho kinase II inhibitor Fasudil, and GFAP expression correlates with expression of NF-κB, indicating that the pathway HIF-1α/Rho kinase II/NF-κB/GFAP is in operation.

**Figure 1:**
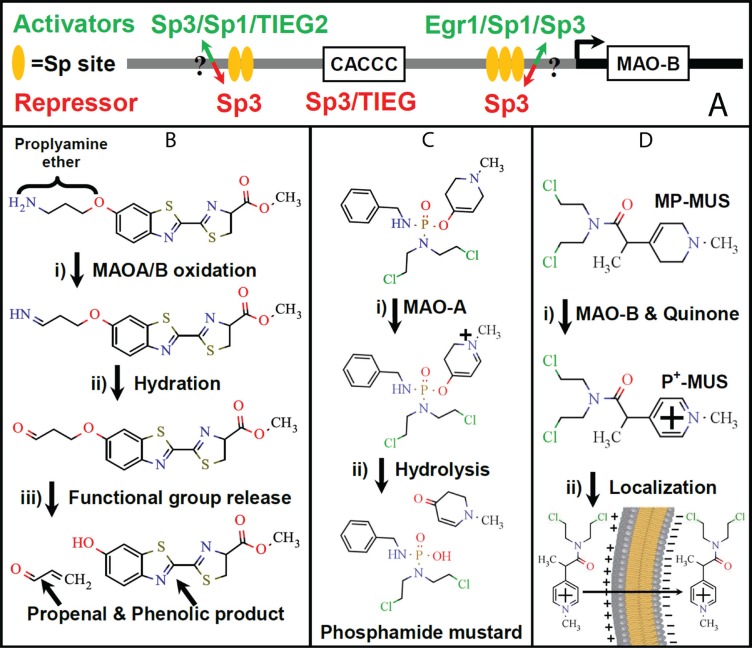
The MAOB gene promoter region and potential MAOB catalyzed prodrug maturation reactions (**A**) The regulation sites of the MAOB gene promoter are shown in cartoon form. There are three main reactions that can use MAOA or MAOB for the conversion of pro-drugs/dyes into their mature, active, form, in the intramembrane space of mitochondria: (**B**) Propylamine ethers undergo oxidation by MAO into the corresponding imine, which then undergo hydration and conversion into the unstable aldehyde. Electron redistribution cleaves the ether bond forming an alcohol and propeneal. The example shown is of the luciferin propylamine ether that is the central component of the MAO-Glo^™^ Assay (Promega). (**C**) 4′ether, N-methyl, tetrahydropyridines undergo oxidation first by MAO, then by quinone, to generate a hydrolysable pyridinium species. The Castagnoli laboratory developed a MAOA specific chemotherapeutic using phosphodiamidate ether with a bis-chloroethyl nitrogen group [35]. (**D**) The conversion of MP-MUS, a tetrahydropyridine, into the stable pyridinium lipophilic cation is catalyzed by MAOB, but not MAOA, in the presence of quinone. The generated P^+^−MUS is electrophoretically concentrated into the mitochondrial matrix by ≈1,000 fold by the mitochondrial membrane potential [36, 37].

There are no HIF-1α binding sites in the promoter region of the human MAOB gene, instead there are a pair of clustered transcription factor Sp1/Sp4/Sp3 binding sites and these are separated by a Sp3/TIEG2 binding site (transforming growth factor β-inducible early gene), CACCC element, Figure 1A. Control of MAOB transcription by Sp1 and Sp3 has been investigated by Wong and co-workers, and generally, Sp1 upregulates and Sp3 inhibits MAOB synthesis [26, 27]. The binding of either TIEG2 or Sp3 to the CACCC element represses MAOB transcription and binding of Sp1 or Sp4 to the Sp-binding sites increases transcription [28]. Guan and co-workers showed that Sp1 levels are high in glioma, and that Sp1 levels are correlated with tumor grade, and inversely correlated to patient survival [29]. An alteration in the levels of Sp1 and Sp3 could link MAOB and HiF-1α levels to tumor grade and aggressiveness.

The degree of MAOB upregulation in glioma cells may be a key to future chemotherapeutics. The rationale for the development of chemotherapeutic prodrugs relies upon the delivery of higher concentrations of a mature drug in target cells compared to non-cancerous tissues. Prodrugs that are enzymatically activated into anti-cancer agents by enzymes that are highly expressed in cancer cells have been developed in the past two decades [30, 31]. As MAOA and MAOB have differently sized and shaped substrate channels leading to the active site, one can design substrates which have isoenzyme-specific activity. A number of MAO isoenzyme-specific substrates have been developed that undergo cleavage following MAO-catalyzed oxidation and release a drug-sized moiety. MAO's have the ability to catalyze three well-explored prodrug maturation mechanisms that release an active drug compound/reporter molecule, Figure 1B–1D. The first uses propylamine ethers as MAO substrates and propylamine-reporter molecule conjugates as the basis of MAO assay systems. In the MAO-Glo^™^ Assay (Promega Corporation, Madison, WI), the substrate is a propylamine ether that is cleaved, releasing luciferin following MAO oxidation [32, 33], Figure 1B. A similar fluorescence-based assay uses the propylamine ether of resorufin as a substrate [34]. The second approach uses a 4'ether or 4'carbamate derivative of a N-methyl, tetrahydropyridine and was pioneered by the Castagnoli laboratory, the same group that developed a MAOA specific chemotherapeutic using a N-methyl-tetrahydrpyridine phosphodiamidate ether with a bis-chloroethyl nitrogen group [35]. This pro-drug is oxidized by MAOA to the dihydropyridine, that then undergoes spontaneous hydrolysis producing the mature, active, chemotherapeutic and methyl-tetrahydropyridinone, Figure 1C. We have recently developed a MAOB specific pro-drug, MP-MUS, that has been designed to target mitochondrial DNA upon maturation and has far higher toxicity toward gliomal cells than toward normal human astrocyte. We have also shown that our lead compound, MP-MUS, can successfully treat primary human glioma xenografts in both flank and intracranial nude mouse models [36, 37]. The final mature drug compound formed by the action of mitochondrial MAOB is the lipophilic cation, P^+^-MUS, which partitions into mitochondria, driven by the mitochondrial ΔΨ, to concentrations approximately 1000 times greater than that present in the intermembrane space. The high concentrations of P^+^-MUS damage the mitochondria, including its DNA, and mitochondrial dysfunction leads to gliomal cell death, Figure 1C.

In this study, we generated a tissue microarray from 20 GBM tumors, nine low-grade astrocytomas, and temporal lobe control brain tissue. We used immunohistochemistry to examine the levels of MAOB, glial fibrillary acidic protein GFAP, HiF-1α and transcription factors Sp1 and Sp3. We sought to confirm previous finding that MAOB levels are higher in GBM and low-grade astrocytomas than in control brain tissue [14], to shed light as to why MAOB is upregulated in these cancers and to determine if the design and use of MAOB specific prodrugs is an appropriate route for glioma chemotherapy.

## RESULTS

### Immunohistochemical labeling of MAOB, HiF-1α, GFAP, Sp1 and Sp3 in gliomas

**Figure 2:**
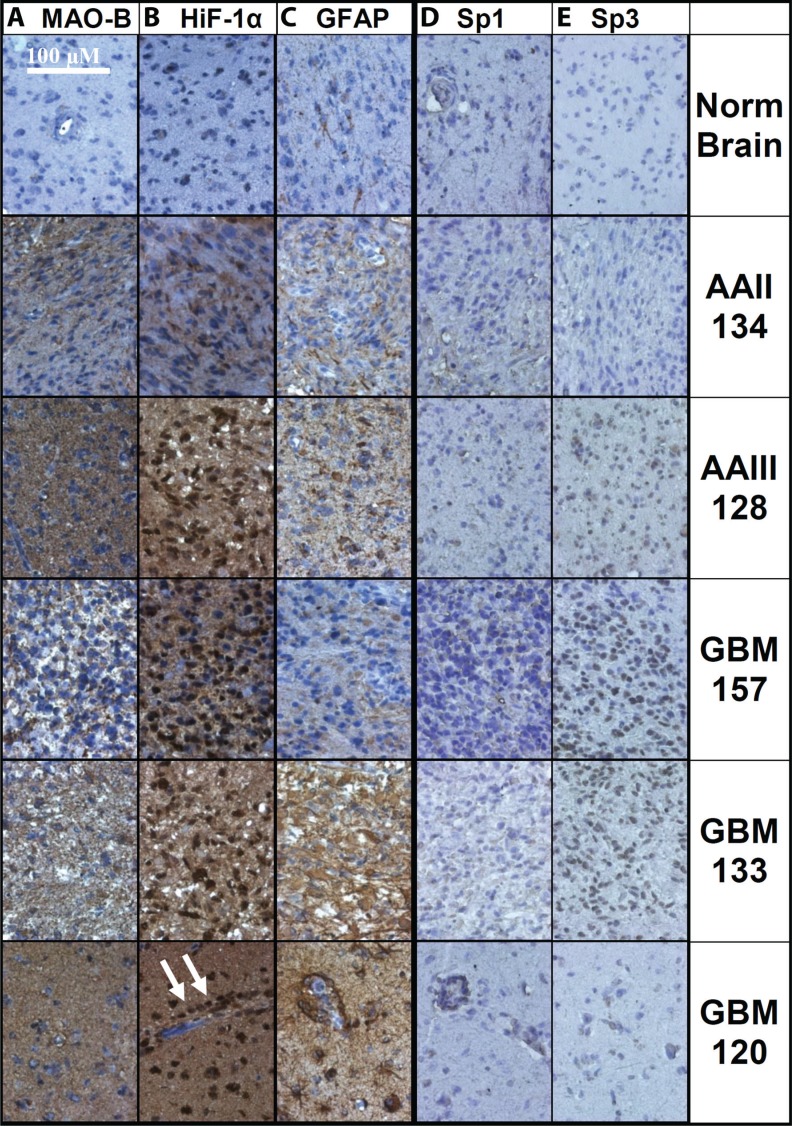
Immunohistochemical labeling of normal brain and glioma tissue Representative images of normal temporal lobe, anaplastic astrocytoma grade II and grade III (AAII and AAIII), and three GBMs with low, medium, and high labeling of (**A**) MAOB, (**B**) HiF-1α, and (**C**) GFAP are shown, along with (**D**) Sp1 and e) Sp3. The antibodies were detected with DAB and the nuclei were labeled with hematoxylin. Each image in a column was taken from a single slide, where each tissue slice was treated identically and imaged using the same microscope setting and with no electronic image manipulation. Arrows indicate nuclear HiF-1α.

Representative images of MAOB, HiF-1α, GFAP, Sp1 and Sp3 immunohistochemical labeling in gliomas and normal brain tissue are shown in Figure 2. Three slides, bearing 20 GBM, 7 lower grade glioma and control human temporal brain tissue, were simultaneously prepared for MAOB, HiF-1α, GFAP immunohistochemical labeling/staining and were imaged in a single session, using identical microscope settings. The slides used for determining the levels of Sp1 and Sp3 were performed in parallel and are directly comparable to each other. MAOB, HiF-1α, and GFAP are far lower in the control tissue (top row) than in any of the GBM (bottom three rows). In a representative grade II astrocytoma (second row), the levels of all three proteins were elevated with respect to the control. Although HiF-1α was elevated, very little of the immunohistochemical labeling was nuclear. In a representative grade III astrocytoma (third row), there were higher levels of all three proteins than in the grade II sample. Additionally, > 80% of the grade III cell nuclei had high expression of HiF-1α. Three representative GBM tumors are shown, which have, respectively, the lowest (GBM157), average (GBM133), and highest (GBM120) levels of immunohistochemical labeling for MAOB, HiF-1α, and GFAP of all 20 GBM tumors examined. In GBM157, MAOB, HiF-1α, and GFAP expression was highly heterogeneous, and HiF-1α was localized to the nucleus in only half the cells. GBM133 had elevated MAOB, HiF-1α, and GFAP expression and > 85% of the nuclei were positive for HiF-1α. GBM120 had the highest relative expression levels of all three proteins with homogenous expression of MAOB as well as diffuse cytosolic expression and regions of intense nuclear staining for HiF-1α. HiF-1α labeling was intense in cells both far from and adjacent to large blood vessels, the latter of which is unlikely to be subjected to hypoxia [38]. GFAP expression co-localized with HiF-1α with high levels in the glioma cells surrounding the blood vessels. On first impression there is little correlation between changes in relative Sp1 or Sp3 levels, and the localization in the cytosol/nucleus and the levels of MAOB. However, in Supplementary Figure 1 we show representative images of Sp3 levels in glioma tumors, without hematoxylin, that we have divided into two populations; Supplementary Figure 1A, where Sp3 immunohistochemical labeling is shown in 16 GBM, arranged in order of increasing MAOB levels and Supplementary Figure 1B, where Sp3 labeling is shown in 4 GBM, 7 astrocytic glioma and a brain section control, again arranged with respect to increasing MAOB levels.

### Quantification and correlation of MAOB, HiF- 1α, and GFAP levels in gliomas

**Figure 3:**
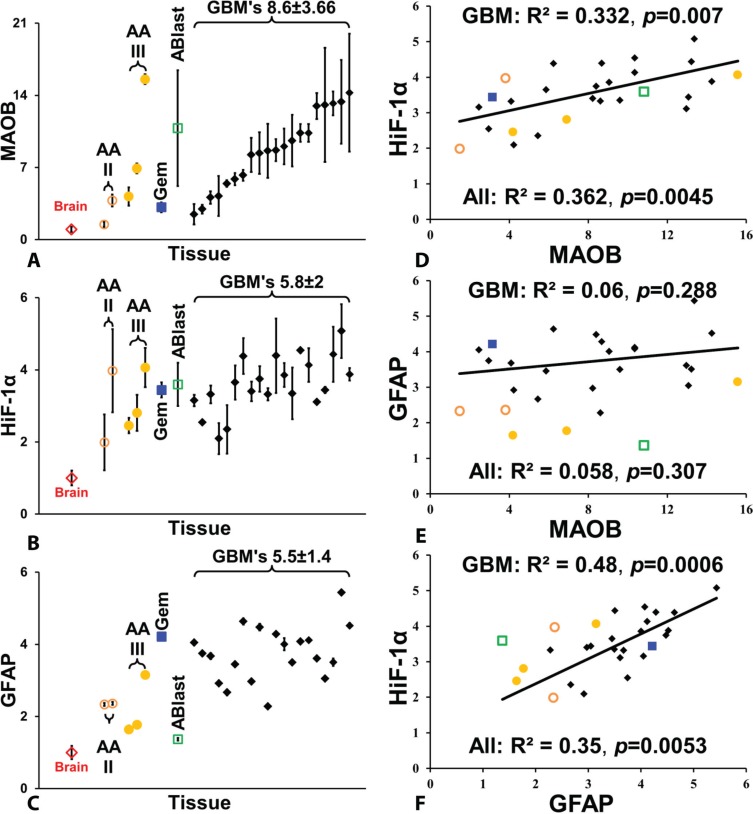
Quantification of MAOB, HiF-1α, and GFAP levels in gliomas (**A**) Relative levels of MAOB in normal brain, five anaplastic astrocytomas (AA), a geministic astrocytoma (Gem), an astroblastoma (Ablast), and 20 GBM tumors, with the GBM tumors arranged from lowest to highest signal. The same left-right arrangement of GBM tumors is also used in (**B**) and (**C**). (B) Relative levels of HiF-1α. (C) Relative levels of GFAP. (**D**) Correlation between MAOB and HiF-1α in gliomas and GBMs. (**E**) Correlation between MAOB and GFAP in gliomas and GBMs. (**F**) Correlation between HiF-1α and GFAP. The coefficient of determination, R2, and *p* values are shown on all correlation plots. All points are mean ± SD, with *n* = 4 for tumor samples and *n* = 12 for the control brain samples.

The quantified levels of MAOB, HiF-1α, and GFAP in all the astrocytoma and GBM samples, relative to control brain tissue, are shown in Figure 3A–3C. The MAOB level of two grade II astrocytomas was greater than 2.5 times that of the control (Figure 3A). The higher grade anaplastic astrocytomas had greater than eight times the MAOB level of the control, with AA(III)128 having the highest level of MAOB among all the gliomas examined. The gemistocytic astrocytoma had MAOB levels similar to the two grade II astrocytomas. The astrocytoblastoma was highly heterogeneous with respect to MAOB, ranging from 4 to 13 times greater than control levels. The 20 GBM tumors are shown in MAOB rank order in Figure 3A, with MAOB levels of 8.6 ± 3.6SD greater than control tissue. The distribution of MAOB in the GBM tumors appears to display a step-function, falling into a three-thirds distribution: 4×, 8×, and 12× greater than the control. HiF-1α levels follow a similar profile to changes in MAOB, with an average upregulation in GBM of 3.6 ± 0.77 times the control level and a similar step-wise trend (Figure 3B). While GFAP levels were also upregulated in all of the GBM (3.75 ± 0.75 times the control level), expression does not appear to correlate directly with MAOB (Figure 3C).

We also compared the cross-correlation of MAOB, HiF-1α and GFAP levels among all the gliomas (Figure 3D–3F). Expression of MAOB and HiF-1α is highly correlated, with a correlation coefficient (R^2^) of 0.33 (*p* = 0.0072) for GBM alone and 0.36 (*p* = 0.0045) in all the glioma (Figure 3D). While both MAOB and GFAP were upregulated, with respect to control brain tissue, there is no significant correlation between these two proteins in the glioma (Figure 3E). Finally, levels of GFAP and HiF-1α are highly correlated, generating a R^2^ value of 0.48 (*p* = 0.0006) in GBM and 0.35 (*p* = 0.0053) in all glioma (Figure 3F). This suggests that MAOB and GFAP levels are under the control of HiF-1α, but that the signaling pathways differ.

### MAOB and peroxide generation in glioma cells and in NHA

**Figure 4:**
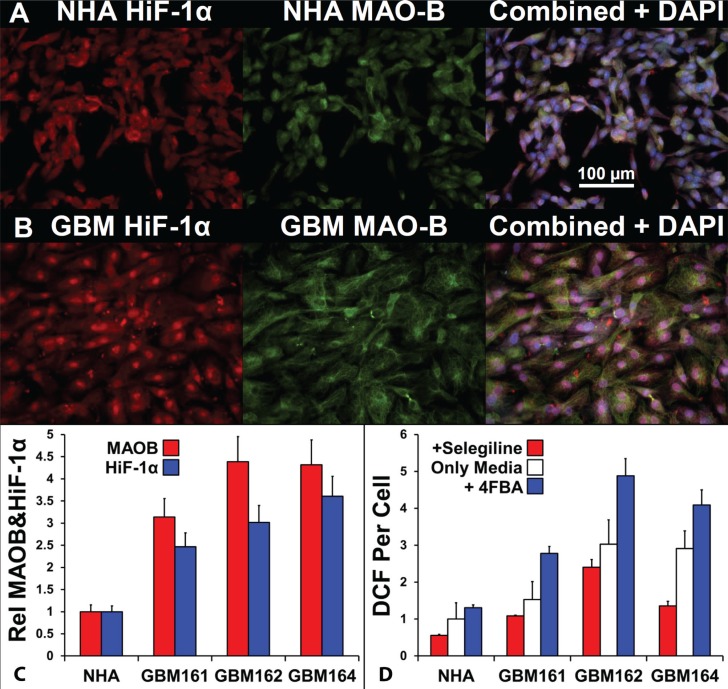
Comparison of MAOB expression, HiF-1α expression, and peroxide generation in response to a MAOB substrate in normal human astrocytes and glioma cells HiF-1α (i, red), MAOB (ii, green), and superimposed HiF-1α/MAOB signals with blue DAPI labeling (iii) of NHAs (**A**) and GBM cells (**B**). (**C**) Relative expression of HiF-1α (white bars) and MAOB (black bars) in NHAs and in three GBM low-passage cultures, *n* = 6, mean ± SD. (**D**) MAOB inhibited (gray bars), endogenous (white bars) and 4-FBA-stimulated (black bars) reactive oxygen species (hydrogen peroxide) generation, shown as DCF per cell, in NHA and the three GBM cultures, *n* = 16, mean ± SD.

We examined the levels and localization of HiF-1α and MAOB in primary, low-passage, human glioma cells, grown at ambient oxygen levels, with normal human astrocyte (NHA), Figure 4. HiF-1α and MAOB levels were higher in the representative GBM (GBM 161, Figure 4B) than in NHA (Figure 4A). Furthermore, the distribution of HiF-1α differed between the two cell types, with a nuclear:cytosolic ratio for HiF-1α of 1.35 ± 0.51 in NHA and 2.6 ± 0.27 in GBM161 (*n* = 10; center fields from 10 individual wells). When we quantified the levels of HiF- 1α and MAOB in three glioma cell cultures with respect to NHA, and the data in Figure 4C indicates a three-fold and four-fold increase in HiF-1α and MAOB levels, respectively (Figure 4C).

To measure the activity of MAOB, the three glioma and NHA cell cultures were incubated with the oxidant sensitive probe, H_2_-DCF-AM, in the absence and presence of the MAOB specific inhibitor Selegiline or substrate, 4-fluorobenzylamine (4-FBA) [39]. After deesterification, the internalized H_2_-DCF probe is oxidized by oxidants, principally peroxide, to the fluorophore DCF [40]. The selegiline sensitive, MAOB specific, rate of DCF generation in NHA is 44% of the background. Addition of the MAOB substrate 4-FBA to NHA results in a 30% increase DCF generation. Thus, in NHA, MAOB is operating at 60% of its maximal flux and this generates almost half the cells hydrogen peroxide production. Preincubation with selegiline revealed that peroxide generation via MAOB was 20% of the steady state level in GBM162, 30% in GBM161and 50% in GBM164. However, in these glioma MAOB flux was much more tightly constrained by MAOB substrate availability, and on addition of 4-FBA these cells generate between 4 and 6 fold more peroxide than do NHA (Figure 3D). The difference between the 4-FBA stimulated and selegiline inhibited rates indicate that the glioma have between 2.3 and 3.7 times more functional MAOB than do NHA. From a MOAB specific pro-drug design perspective, these data show that the coupling of HiF-1α and MAOB, and the elevation of both in GBM, is not dependent of oxygen tension.

### Quantification and correlation of Sp1 and Sp3 levels in gliomas

**Figure 5:**
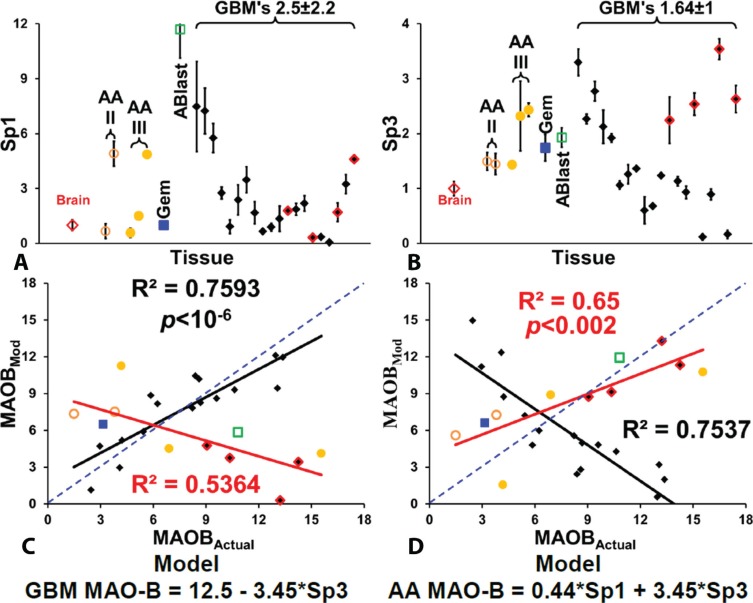
Quantification of Sp1 and Sp3 levels in gliomas Relative levels of Sp1 (**A**) and Sp3 (**B**) in normal brain, five anaplastic astrocytomas, a geministic astrocytoma, an astroblastoma, and 16 GBM tumors, with the GBM tumors arranged from lowest to highest signal. In the case of Sp3 the four GBM that appear as outliners are indicated using red-lined diamond symbols. The modeling of the levels of MAOB, based on Sp1 and Sp3 levels is show for the 16 GBM in (**C**) and for the astrocytic-type glioma and 4 aberrant GBM in (**D**). All points are mean ± SD, with *n* = 4 for tumor samples and *n* = 12 for the control brain samples.

We quantified levels of transcription factors Sp1 and Sp3 in the astrocytoma and GBM samples, relative to control brain tissue, and these are shown in Figure 5A and 5B, with tissue sample arranged as in Figure 3, so the GBM's with the lowest level of MAOB are on the left and highest levels on the far right. An examination of the levels immediately reveals two properties, firstly that levels of Sp1 and Sp3 are not at all similar to levels of MAOB, HiF-1α or GFAP. We modeled the relationship between MAOB levels and Sp1 and Sp3 for the GBM and non-GBM populations and found that we could discern two general relationships, and that if we treated four of the GBM's as astrocytic glioma, the relationship became more pronounced; the 4 GBM's that appear to be more typical of astrocytic glioma than the general GBM population are shown in Figure 5A–5D as red highlighted black diamonds. The level of MAOB in the 16 GBM fits a model whereby maximum expression, 12.5 times greater than normal brain tissue, is repressed by Sp3, so that MAOB levels are inversely proportional to Sp3 levels. The fit is shown in Figure 5C, with the blue line representing a perfect, unity, fit. The fit of the modeled MAOB to actual measured MAOB levels, based only on Sp3, is very good. What Figure 5C also shows is that the same fitting algorithm that produces a positive correlation for the 16 mainstream GBM also produces a very good correlation (R^2^ > 0.5) for the astrocytic glioma and the four aberrant GBM. However the slope of the fit is inversed in the two groups of tumors; that is, MAOB positively correlated with Sp3 levels in the astrocytoma and aberrant GBM and is negatively correlated as in the case of the 16 mainstream GBM, hence the opposing slopes of the black and red fitted lines. Figure 4E shows the best modeled fit to the astrocytic glioma and the 4 recalcitrant GBM. In these cells MAOB levels are tightly coupled to both Sp1 and to Sp3, and in both cases the relationship is positive.

The failure of fit, both from unity and from the fitted line, is partly due to the localization of Sp3 within the tumor cells. In both Supplementary Figure 1A and 1B it is evident that there are two different trends in the cellular localization of Sp3 in the glioma cells, with respect to rising MAOB.

In the 16 GBM where Sp3 acts as a repressor of MAOB expression, as MAOB levels increase there is a loss of Sp3 label from the cell nucleus. The GBM with the lowest level of MAOB, GBM157, has highly Sp3-labeled nuclei and little Sp3 in the cytosol whereas GBM115, which has the highest level of MAOB expression, has no discernible Sp3 stained nuclei, Supplementary Figure 1A.

In the case of the astrocytic glioma, and the four GBM's that appear to fit with them, where our modeling studies suggest that SP3 is a MAOB promoter activator, we find the opposite relationship between localization and MAOB levels. In these tumors, low levels of Sp3 in the nuclei correlate with low MAOB levels, and where we find MAOB levels, we find nuclear Sp3.

Finally, in Figure 3 we noted that there was very poor correlation between MAOB and GFAP, but noted that GFAP and HiF-1α were well correlated. We modeled the levels GFAP, floating all the other measured protein levels and find that we can get a very good estimate of GFAP levels in the 16 GBM, but not the astrocytic group of glioma, using three parameters, HiF-1α, Sp1 and Sp3, all of which are positive, Supplementary Figure 2. We were unable to derive a reasonable fit, using up to four parameters (MAOB, HiF-1α, Sp1 and Sp3) to model GFAP in the astrocytic cells, which is in conflict with Professor John von Neumann's assertion on the nature of multi-parameter models [41].

## DISCUSSION

The results of this study demonstrate that the levels of MAOB were, on average, 8 times higher in gliomas than in control tissue, which is higher than the activity levels previously described [14]. The upregulation of MAOB was also correlated with an increase in HiF-1α. In cancers, the active form of HiF-1α is responsible for shifting cellular metabolism from aerobic to anaerobic (i.e., the “Warburg Effect”) and for increasing vascularization through the upregulation of angiogenic proteins [42]. Vascular Endothelial Growth Factor-A (VEGF-A) [43], GFAP [18] and muscle-specific pyruvate kinase-M2 [44] (PKM2) are upregulated in glioma, and the level of upregulation of VEGF, VEGF receptor 2 (VEGFR2) [45], GFAP [19], and PKM2 [46] correlate with tumor grade and lethality (review [47]). The promoter regions of VEGF-A and PKM2 have a number of Sp1/Sp3 activator/repressor elements that control transcription levels of these genes. Hypoxia has been shown to deplete Sp3 binding, leading to the reactivation of Sp3-repressed genes [48]. We find that in the majority of the 20 GBM's analyzed MAOB is tightly coupled to the levels of Sp3, which is acting as a repressor in these cells.

The genes encoding GFAP and MAOB also have Sp1/Sp3 domains in their promoter regions, and increasing Sp1 or decreasing Sp3 levels leads to an increase in MAOB expression [26, 27, 49].

MAOB generates hydrogen peroxide, and we found high basal rates of reactive oxygen species generation, mostly hydrogen peroxide, in glioma cells as compared to NHA. Incubation with a MAOB substrate elevated the peroxide generation to an even greater extent. Hydrogen peroxide has been shown to increase Sp1 levels [50] and decrease Sp3 levels [51], and the peroxide-induced alteration of the Sp1:Sp3 ratio leads to an increase in HiF-1α [52]. In normal cells, at physiological oxygen levels, the majority of HiF-1α is in an inactive form and incapable of binding HiF-1α DNA targets. Factor Inhibiting HIF- 1 (FIH-1) catalyzes the hydroxylation of a specific asparagine residue on HiF-1α, which blocks HiF-1α's transcriptional activity [53]. FIH-1 activity toward HiF- 1α is oxygen dependent, and under normoxic conditions FIH- 1 quickly inactivates HiF-1α. However, FIH-1 is very sensitive to peroxide denaturation [54], and relatively low levels of peroxide inhibit FIH-1, leading to a withdrawal of HiF-1α inactivation. Additionally, it has been shown in that reactive oxygen species upregulate HIF-1α via the binding of NF-κB, which is oxidant dependent [55]. In glioma, and perhaps other cancers [56], we suggest that the high steady state peroxide levels inhibit FIH-1, increasing both total and active HiF-1α, which we found to be localized to the nucleus. Furthermore, high levels of active HiF-1α, shifts the cellular metabolism from aerobic to anaerobic. Therefore, the upregulation of MAOB appears to be tied to a positive feedback signaling loop, where MAOB increases peroxide levels, peroxide facilitates Sp3 repression and upregulation HiF-1α and Sp1, and these events ultimately increase MAOB expression.

A very similar function for MAOA in prostate cancer has been recently suggested by Wu and co-workers [57]. They find that MAOA generated H_2_O_2_ to induce epithelial-to-mesenchymal transition via stabilization of HIF-1α. This peroxide-induced stabilization of HIF-1α increases proliferation, invasiveness, and metastasis of prostate cancer cells.

There is an apparent paradox as to the nature of Sp3 and its causing upregulation of MAOB levels in low grade astrocytic glioma and its acting as a repressor in of MAOB transcription in GBM. However, the Sp3 gene has four start codons that allow the generation of four different protein products [58], with different properties, and these variants are all subject to functional sculpting via post-transcriptional modifications that include, phosphorylation, acylation and SUMOation [59, 60]. Like the changes on GBM herein, Wong found that Sp3 repressed MAOB transcription [27] in Caco-2 cells, but also showed that DNA-methylation fine-tuned MAOB levels by altering the affinity of Sp1/Sp3. Sp3 activity has been shown to be regulated by SUMO-1 modification, and such modification can flip its activity from being a repressor to activator, and vice versa. Native, unSUMOylated, Sp3 generally acts as an activator at its promoter sites, and nuclear localization is diffuse, with Sp3 binding to many promoter elements scattered throughout the genome. Following SUMOylation the SUMO-Sp3 is found bound to DNA at the nuclear periphery and inside the nucleus in small clusters and inversion occurs and much Sp3-dependent transcription is repressed [58, 61–64]. This inversion of function, with Sp3 repressing MAOB transcription in GBM and activating it in the astrocytic glioma, may be linked to SUMOylation changes in these glioma, and such a change appears to be linked to tumor grade. SUMOylation changes in proteins, and on the expression of HiF1-a promoters have been shown to be dependent on Sentrin/SUMO-specific protease3 (SENP3) [65]. The de-SUMOylating activity of SENP3 is increased by modest levels of peroxide and SENP3 deSUMOates p300, a HIF-1α transactivator.

**Figure 6:**
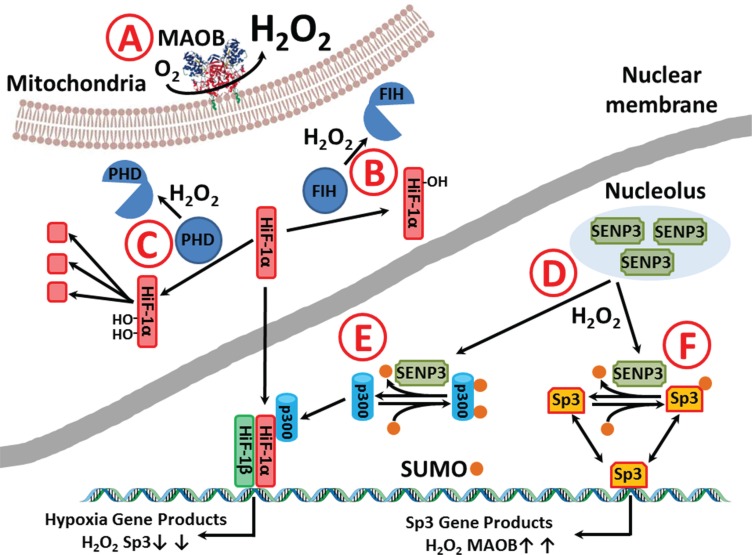
Putative MAOB alteration in gliomal phenotype (**A**) Hydrogen peroxide generated by MAOB (A) alters the fate of HIF-1α vie two distinct mechanisms. (**B**) FIH which normally inactivates HIF-1α under normoxic conditions is inactivated by peroxide. (**C**) The degredation of HIF-1a is dependent on PHD modification that initiates ubiqutination and preoteolysis and PHD's are peroxide sensitive. (**D**) Peroxide causes SUMO-specific proteases 3 (SENP3) migration from the nucleolus and causes the deSUMOylation of regulatory proteins. (**E**) The deSUMOylation of p300 allows the active HIF-1α/HIF-1α /p300 to bind to hypoxia response elements and increase transcription of hypoxia induced proteins, like VEGF. (**F**) SENP3 may be able to deSUMOylate Sp3 altering the balance between activation/repression of Sp3 at Sp3 binding site, including the repressor elements in the MAOB promotor, so that an increase in deSUMOylation causes an upregulation of MAOB levels, hence a further increase in cellular peroxide.

Figure 6 shows a graphical representation of how we believe that elevated MAOB drive increased cellular peroxide levels in GBM and initiates the ‘Warburg’ phenotype. Hydrogen peroxide generated by MAOB (A) is freely diffusible and can alter the fate of HIF-1α vie two distinct mechanisms. Firstly, FIH, which irreversibly modifies and inactivates HIF-1α is highly sensitive to peroxide inhibition [53, 54] and modest increases in ROS will extend the life of active HIF-1a (B). The degredation of HIF-1a is dependent on proline hydroxylases (PHD's) that are in turn deactivated by peroxide (C) [66, 67]. SUMO-specific proteases 3 (SENP3) is capable of deSUMOylation of a range of SUMOylated protines, including p300. Nuclear levels of SENP3 are modulated by ROS, with delocalization from the nucleolus observed following increased peroxide levels(C) [65, 68–70]. SENP3 regulates the transcriptional activity of HIF- 1α via deSUMOylation of the coregulatory protein p300, with elevated peroxide increasing levels of the active HIF-1α/HIF-1α/p300 complex (D) [69]. Sp3 is functionally altered by SUMOylation [64] and so putative SENP3 deSUMOylation of Sp3 will alter the balance of Sp3:SUMO-Sp3 in favor for the former (D). In GBM this alteration in Sp3 resulting in an upregulation of MAOB, and so leads to a further increase in peroxide levels. Such an alteration in the balance of SUMOylation pathway would not only explain why GBM grown at atmospheric oxygen tensions still display high levels of HIF-1α and why this HIF-1α is located in the nucleus, Figure 4, but why the relationship between the levels of MAOB and Sp3 in the bulk of the GMB is inverted in the astrocytoma and in four of the GBM tumor, Figure 5.

In conclusion, we confirmed that MAOB is highly expressed in glioma and found that the highest grade tumors have the highest expression levels of MAOB. This increased expression, along with the ability of MAOB to catalytically mature, active, chemotherapeutic prodrugs, offers a new route to gliomal therapy.

We are currently investigating a number of MAOB sensitive pro-drugs, such as MP-MUS, that may be useful in the treatment of cancers that overexpress MAOB.

## MATERIALS AND METHODS

### Tissue and cells

Following signed informed consent and institutional review, tissue samples were obtained from patients undergoing surgically indicated resection of malignant gliomas at the Houston Methodist Hospital. Tissue was collected directly from the operating room, given an identifying code to ensure patient confidentiality, and fixed in ice-cold 4% paraformaldehyde (PFA) within 15 minutes of removal. In one of the patients, the tumor location necessitated the removal of a small amount of normal temporal brain tissue, and this was used as our control. After fixation at 4°C for greater than 4 days, the samples were dehydrated in graded ethanol, washed in xylene, then waxed using Paraplast Plus^®^ (Lecia Biosystems, Richmond, IL, USA). Waxed cores 2 mm in size were generated using the Tissue-Tek^®^ Quick-Ray^™^ system (Sakura Finetek USA, Inc. Torrance, CA, USA) according to the manufacturer's instructions. A single core was used for each glioma sample, along with two cores of normal temporal lobe tissue. A second tumor sample from each patient was also characterized by a neuropathologist and classified according to the World Health Organization (WHO) Classification of Tumours of the Central Nervous System (CNS). The tumors were diagnosed as low-grade astrocytoma (WHO grade II; *n* = 2), anaplastic astrocytoma (WHO grade III; *n* = 3), gemistocytic astrocytoma (WHO grade III; *n* = 1), astrocytoblastoma (WHO grade III; *n* = 1), and glioblastoma multiforme (WHO grade IV; *n* = 19).

Additional glioblastoma tumor tissues taken at the time of excision were finely sliced using a scalpel and then homogenized in growth media with a 5 ml pipette. The cells were grown in Dulbecco's Modified Eagle's Medium (DMEM) with Fetal Bovine Serum (FBS, 20%), 1 × GlutaMax-I, sodium pyruvate (1 mM), Penicillin (100 U/ml), and Streptomycin (100 μg/ml). In all three GBM primary cultures reported here, the cells were spontaneously immortalized and were used between the third and fourth passage. Glioma cells were grown to confluency in Costar 96-well plates (Corning, NYC, NY, USA) or 16-well Lab-Tek slide chambers (Nalgene Nunc International, Rochester, NY, USA).

Normal human astrocytes (NHA) were obtained from Lonza (Walkersville, MD, USA) and cultured as recommended. NHAs were grown to confluency in Astrocyte Cell Basal Medium (Lonza) supplemented with 3% FBS, 1% Glutamine, 0.25% Insulin, 0.1% fhEGF, 0.1%GA-1000, and 0.1% Ascorbic acid in 96-well plates or in 16-well Lab-Tek slide chambers in a total volume of 250 μl.

### Histological analysis of tissue and cells

The tissue microarray block was sliced into 5-μm sections that were affixed to slides and dried. Slides were dewaxed four times in xylene, twice in isopropanol, and rehydrated using graded ethanol. The slides were washed and permabilized using Phosphate Buffered Saline (PBS, Fisher Scientific, Waltham, MA) containing 0.1% Triton X-100. Epitope retrieval was performed by heating the slides in Na-citrate buffer (100 mM, pH 6.0) at < 100°C in a domestic vegetable steamer for 30 minutes, followed by cooling slowly to bench temperature. After washing in PBS, endogenous peroxidase activity was eliminated using mild conditions: 1.8% H_2_O_2_ for 5 minutes, 1% Periodate for 5 minutes, 0.02% NaBH_4_ for 2 minutes [71]. The slides were blocked using Serum-Free Protein Block (Dako North America, Inc., Carpinteria, CA, USA) and incubated with primary antibodies overnight at room temperature, at 1:100 dilution. After washing in PBS, the HiDefTM HRP-polymer system (Cell Marque, Rocklin, CA, USA) was used to functionalize with peroxidase, and visualization was performed using the Dako DAB chromogen kit according to manufacturer's guidelines. Slides used for quantification were treated with DAPI and visualized using fluorescence microscopy to identify non-necrotic areas of the tumors. Additional slides were also incubated with hematoxylin/100 mM LiOH to render the nuclei blue for visualization of cells using transmission microscopy.

Glioma cells and NHA were fixed in ice-cold 4% PFA and were washed/permeabilized with 0.1% Triton X-100 in PBS and treated with Dako protein blocking solution. Levels of ketones/aldehyde were measured using ARP/borohydride (Invitrogen/Molecular Probes, Eugene, OR, USA) and visualized using fluorescein isothiocyanate conjugated with egg white avidin (FITC-avidin) as previously described [72].

### Primary and secondary antibodies

We used the following primary antibodies: rabbit monoclonal to Monoamine Oxidase B [EPR7103], mouse monoclonal to GFAP [2A5] (Abcam, Cambridge, MA, USA), mouse monoclonal to HiF-1α (NB100–105; Novus Biologicals, Littleton, CO, USA), mouse monoclonal to Sp3 [D-6] (Santa Cruz Biotechnology, CA) and rabbit polyclonal to Sp1 [sc-59] (Santa Cruz). We used the following secondary antibodies: goat anti-rabbit IgG antibody labeled with Alexa Fluor^®^488 (A-11034) and goat anti-mouse IgG antibody labeled with Alexa Fluor^®^594 [A-11005] (Life Technologies, Carlsbad, CA). All antibodies were diluted using Dako antibody diluent (S3022) at 1:100 for the primary antibodies and 1:250 for the secondary antibodies.

### Measurement of hydrogen peroxide generation in cells

Cells grown in a 96-well format were incubated at 37°C with 10 μM H_2_DCF-AM (D-399; Life Technologies) in 250 μl of 10 mM glucose/3 mM Tris/30 mM HEPES/10 mM NaCl buffer, pH 7.4, in the absence or presence of either the monoamine oxidase B specific substrate, 6 mM 4-fluorobenzylamine (Sigma-Aldrich, St. Louis, MO, USA) or to cells pre-incubated for 60 minutes with 5 μM Selegiline (Sigma-Aldrich). The fluorescent product was measured after 30 minutes incubation with a BioTek Synergy HT spectrophotometer (excitation 485/20 nm, emission 528/20 nm).

### Microscopic quantification of signal

Images of DAB-polymer were acquired using a Nikon Eclipse TE2000-E microscope equipped with a CoolSnap ES digital camera system (RoperScientific, (now Photometrics) Tucson, AZ)) containing a CCD-1300-Y/HS 1392 × 1040 imaging array cooled by Peltier. Images at a magnification of 20× were recorded through a blue filter as high resolution jpeg 2000 files and analyzed using Nikon NIS-Elements software. The transmitted blue light of each image was converted into apparent absorbance. The relative levels of the assayed proteins were calculated as a ratio of the average absorbance of tumor:brain after background signal subtraction. The background absorbance was measured in normal temporal lobe tissue that underwent peroxidase/DAB immunohistochemical labeling in the absence of primary antibody (*n* = 4). Levels of antibody binding to control brain were also measured (*n* = 12). Three unique, nuclei-dense areas from each slide were quantified to control for sample heterogeneity. Ratios are presented as mean ± SD for 12 individual images of control brain and three images from tumor tissue.

Color images were acquired using an Olympus IX71 microscope equipped with an Olympus DP72 camera and illuminated using an Olympus TH4–100 light source. Images were recorded using DP2-BSW software (Olympus) and were stored in jpeg format.

Images of fluorescently labeled antibodies were captured using a Nikon Eclipse TE2000-E at 20 × magnification with cell nuclei visualized using DAPI (D1306; Invitrogen). Images presented in Figure 4A and 4B were acquired under the same microscope settings, as were the images used to quantify signal levels in Figure 4C.

### Modeling MAOB and GFAP levels in cells

Modeling studies were performed using Excel, using the solver function to float constants and fitting to the lowest sum of squares. The algorithm was written in the form:

MAOB_model_ = A*Sp1 + B*Sp3 + C*HiF-1α + D

And the output was Sum(MAOB_actual_ - MAOB_model_)^2^. The solver function was used float A, B, C and D to arrive at the smallest sum of squares. Initial fits were performed using two parameters (A and B or A and C) and HiF-1α was only used if a model failed at the *p* < 0.05 level.

## SUPPLEMENTARY MATERIALS FIGURES



## Abbreviations

GBM: Glioblastoma multiforme
AA: anaplastic astrocytoma
Gem: gemistocytic astrocytoma
Ablast: astrocytoblastoma
MGMT: *O*^6^-methylguanine DNA methyltransferase
MAOA and MAOB: Monoamine oxidase A and B
HiF-1α: hypoxia-inducible factor 1-alpha
GFAP: glial fibrillary acidic protein
FIH: factor inhibiting HIF
PHD's: proline hydroxylases
NHA: normal human astrocytes
4-FBA: 4-fluoro-benzylamine
MP-MUS: N,N-bis(2-chloroethyl)-2-(1-methyl-3,6-dihydro-2H-pyridin-4-yl)propanamide
P^+^-MUS: 4-{1- [bis(2-chloroethyl)carbamoyl]ethyl}-1-methylpyridinium
H_2_-DCF-DA: 2′,7′-dichlorodihydrofluorescein diacetate
