# Supplementary material for: Monoamine oxidase B levels are highly expressed in human gliomas and are correlated with the expression of HiF-1α and with transcription factors Sp1 and Sp3
Source: Oncotarget. 2015 Dec 12;7(3):3379–93. doi: 10.18632/oncotarget.6582 (PMC4823113; doi:10.18632/oncotarget.6582)
Supplement: Supplementary file 1 [file oncotarget-07-3379-s001.pdf]

Monoamine oxidase B levels are highly expressed in human gliomas and are correlated with the expression of HiF-1α and with transcription factors Sp1 and Sp3

Supplementary Materials

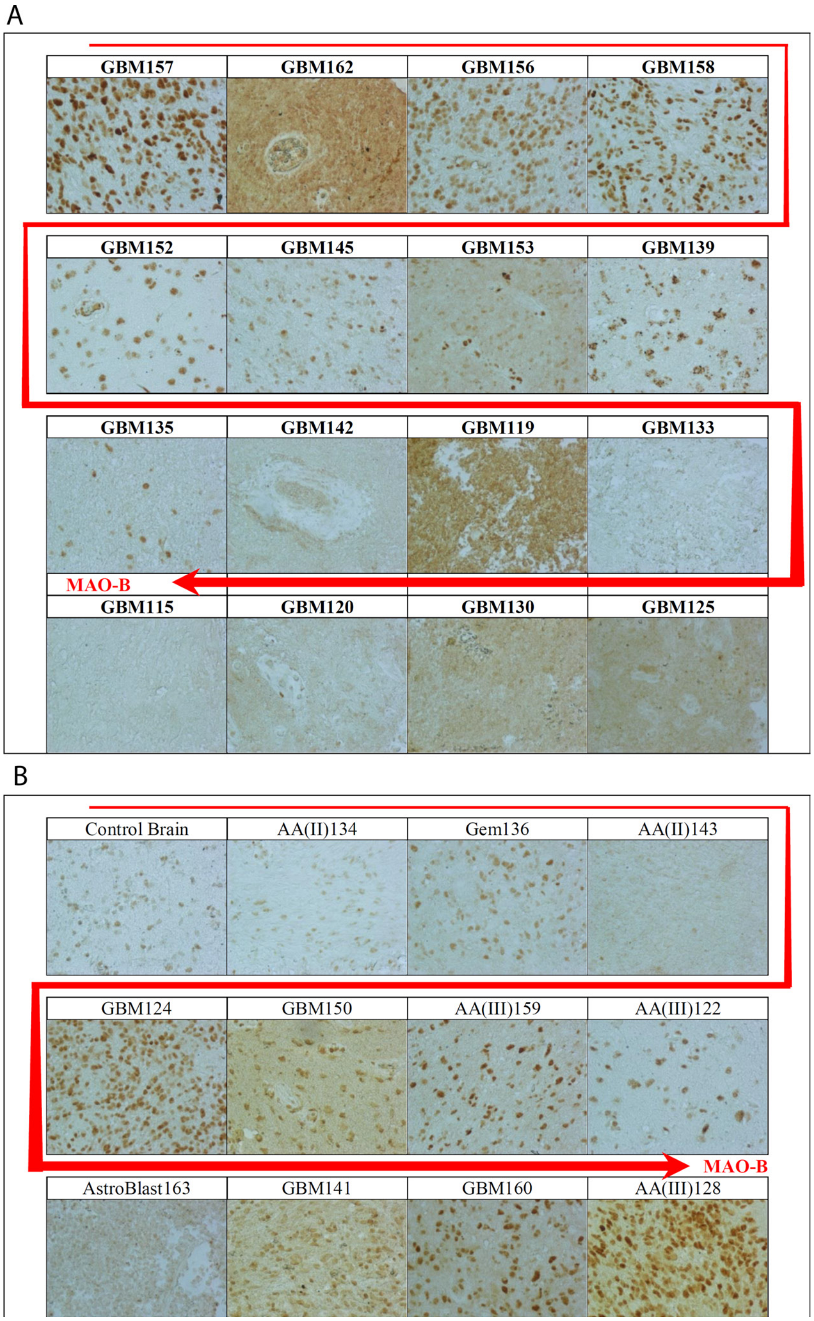

Supplementary Figure S1: Sp3 levels and localization in gliomal microarray. (**A**) shows 16 GBM, arranged in order of their expression of MAOB (low to high), labeled for Sp3. It is noteworthy that high nuclear levels of Sp3 **negatively** correlate with MAOB, and tumor aggressiveness. (**B**) shows control human brain tissue, astrocytoma's and four GBM, arranged in order of their expression of MAOB (low to high), labeled for Sp3. In these tumors high nuclear levels of Sp3 positively correlate with MAOB, and tumor aggressiveness.

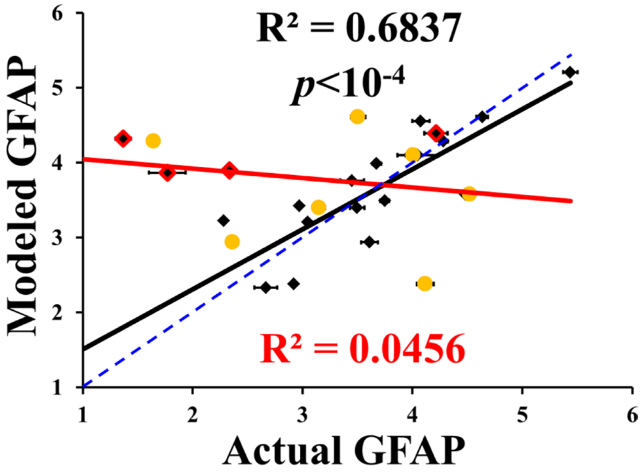

HiF1a Sp1 SP3
0.906 0.123 0.102

Supplementary Figure S2: Modeling of GFAP using HiF-1α, Sp1 and Sp3. Supplementary Figure 2 shows the modeled fit vs. actual measured GFAP in the microarray, with the same symbols used as in Figure 4.
